# Supplementary material for: Ribosome biogenesis as a potential therapeutic target in KRAS mutant colorectal cancer
Source: Nat Commun. 2025 Dec 27;17:1224. doi: 10.1038/s41467-025-67979-9 (PMC12864942; doi:10.1038/s41467-025-67979-9)
Supplement: Supplementary file 1 — Supplementary Information [file 41467_2025_67979_MOESM1_ESM.pdf]

Supplementary Information

Title:

Ribosome biogenesis as a potential therapeutic target in KRAS mutant colorectal cancer.

Authors:

Yui Tanaka[1,2, 10], Mizuho Sakahara[1, 10], Hitomi Yamanaka[1,] Yasuko Natsume[1], Daisuke Kusama[1], Kohei Kumegawa[3], Harunori Yoshikawa[4], Yuich Abe[5,‡], Koji Okabayashi[2], Shimpei Matui[2], Yuko Kitagawa[2], Naohiko Koshikawa[6], Hiroki Osumi[7], Eiji Shinozaki[7], Satoshi Nagayama[8], Jun Adachi[5], Reo Maruyama[3,9] and Ryoji Yao[1, †]

[†]Correspondence may be addressed to ryao@jfcr.or.jp

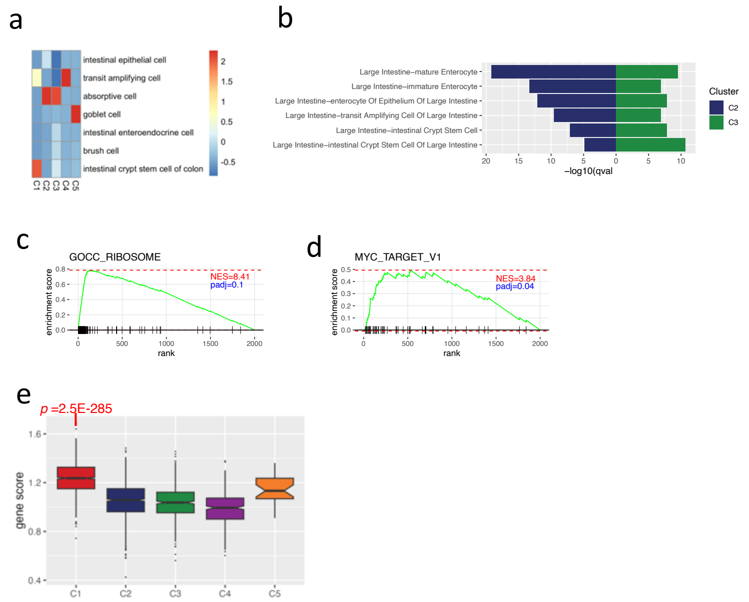

Supplementary Figure 1. Trametinib alters the cellular states of colorectal cancer (CRC) organoids. a. Cell state of CRC patient-derived organoids (PDOs). Single cells in untreated and trametinib-treated PDOs were divided into five clusters using Uniform Manifold Approximation and Projection (UMAP) analysis of single-cell gene expression data and categorized based on the expression profiles of the indicated cell types in the Tabula Sapiens dataset (https://tabula-sapiens-portal.ds.czbiohub.org/). **b.** Molecular characterization of C2 and C3. Enrichment scores for differentially expressed genes in C2 and C3 were calculated using the large intestine gene signatures from the Tabula Sapiens datasets. **c, d.** Gene set enrichment analysis (GSEA) using the RIBOSOME signature in cellular components in the Gene Ontology database (GOCC) (c) and MYC_Target_V1 signature in hallmark gene sets in Molecular Signatures Database (MsigDB). The GSEA-style plot with NES and Padj scores is shown. **e.** Boxplot depicting the different expression levels of gene listed in GOCC_RIBOSOME among clusters. The central line of each box indicates the median, the box bounds represent the interquartile range (IQR), and whiskers extend to 1.5× IQR. P values for C1 and all other comparisons were calculated using a two-sided Welch's t-test.

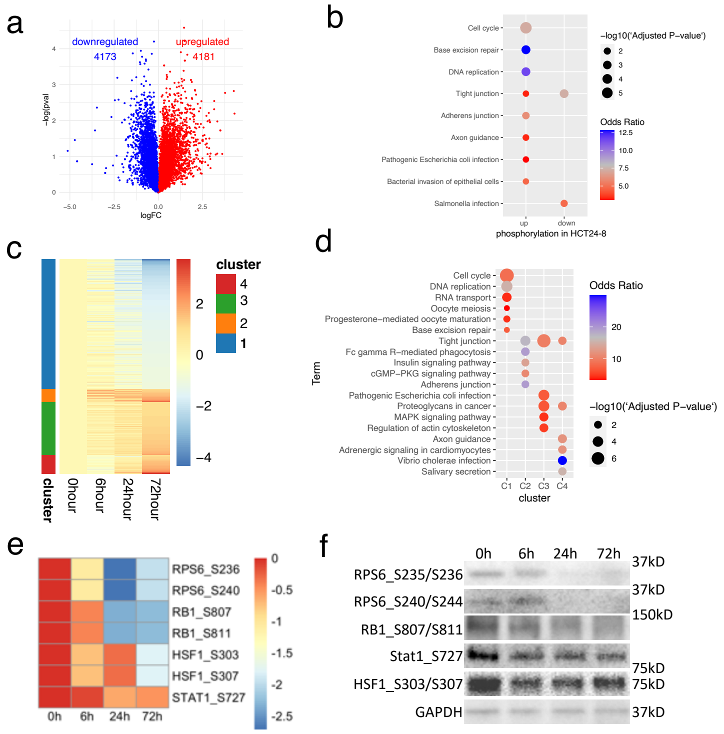

Supplementary Figure 2. Kyoto Encyclopedia of Genes and Genomes (KEGG) signaling pathway phospho-proteomic analysis. a. Volcano plot depicting the distinct phosphorylation patterns between *KRAS*-mutant and wild-type PDOs. Upregulated (red) and downregulated (blue) phosphorylation sites in HCT24-8 are indicated by numbers identified using mass spectrometry. **b.** Enrichment analysis of differentially phosphorylated proteins using KEGG Gene Ontology. Protein phosphorylation is upregulated (up) and downregulated (down) in HCT24-8. **c.** Heatmap showing the time-dependent phosphorylation after trametinib treatment. Four clusters identified via k-means clustering with k=4 are indicated. Color scale on the heat maps depicts the fold-change in phosphorylation. **d.** Enrichment analysis of the differentially phosphorylated proteins. Genes in each cluster identified via k-means clustering (i) were analyzed using KEGG Gene Ontology. **e**. Heatmap showing phosphorylation levels of representative proteins. **f.** Immunoblot analysis of the proteins shown in (e). Phosphorylation at the indicated sites was evaluated using phospho-specific antibodies. GAPDH is shown as the loading control.

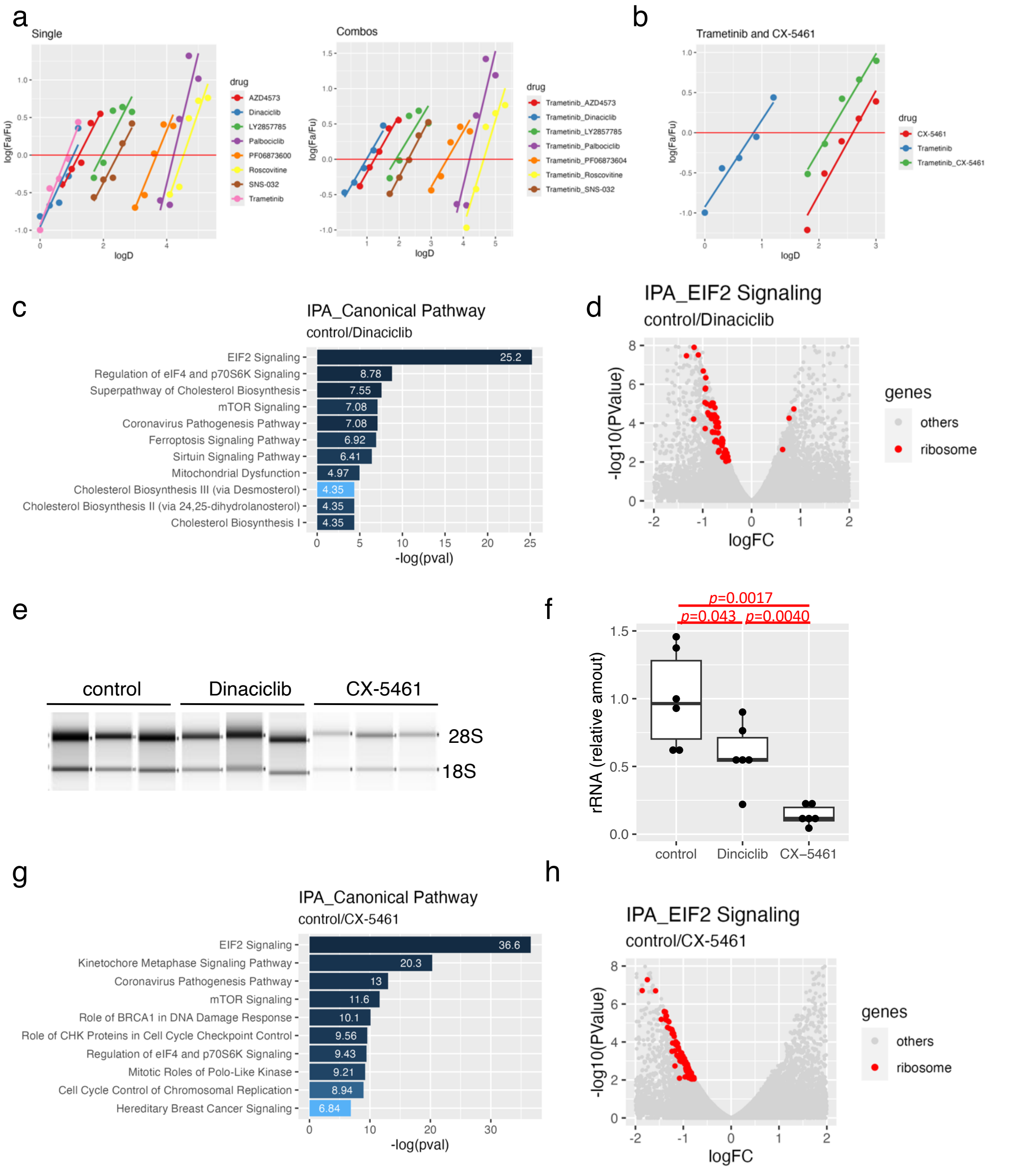

Supplementary Figure 3. Dinaciclib and CX-5461 reduce the expression levels of eukaryotic initiation factor 2 (EIF2) signaling molecules. a,b. Median-effect plot of CDK inhibitors as a single agent (single) or as combination with trametinib (Combos) (a) and of combination of CX-5461 and trametinib (b). The fraction of affected (Fa) versus the drug concentration (D) was plotted in a log-log scale as log(Fa/Fu) versus log(D), where Fu=1-Fa. **c.**Top 10 most significantly enriched signatures in the ingenuity pathway analysis (IPA)

canonical pathway in dinaciclib-treated PDOs. Enrichment scores were calculated using differentially expressed genes (DEGs) identified by comparing the treated and untreated PDOs using Fisher's exact test. **d.** Volcano plot showing the DEGs in trametinib-treated HCT24-8. Vertical axis corresponds to -log10(P value), and the horizontal axis shows the log2 fold change (logFC) value. Red dots represent the EIF2 signaling gene sets in IPA. **e.** Ribosome RNA analysis. HCT24-8 was treated with dinaciclib (10 nM) and CX-5461 (1 μM) for 24 h, and total RNA was extracted and analyzed using Tape Station. The 28S and 18S ribosomal RNAs are indicated. Representative samples from three independent experiments are shown. **f.** Quantification of ribosome RNA. The images in **c** were quantified, and the values relative to the mean intensity of the control are shown as boxplots. Black line in the box plot represents the median value. Bottom and top of the boxplot represent the 25th and 75th percentiles, respectively. *p*-values were determined by two-sided Welch's *t*-test. (N=6) **g.** Top 10 most significantly enriched signatures in the IPA canonical pathway in CX-5461-treated PDOs. The enrichment score was obtained as described in **c**. **h.** Volcano plot showing the DEGs in HCT24-8 between the control and CX-5461-treated HCT24-8. The p and logFC values were calculated as described in **d**.

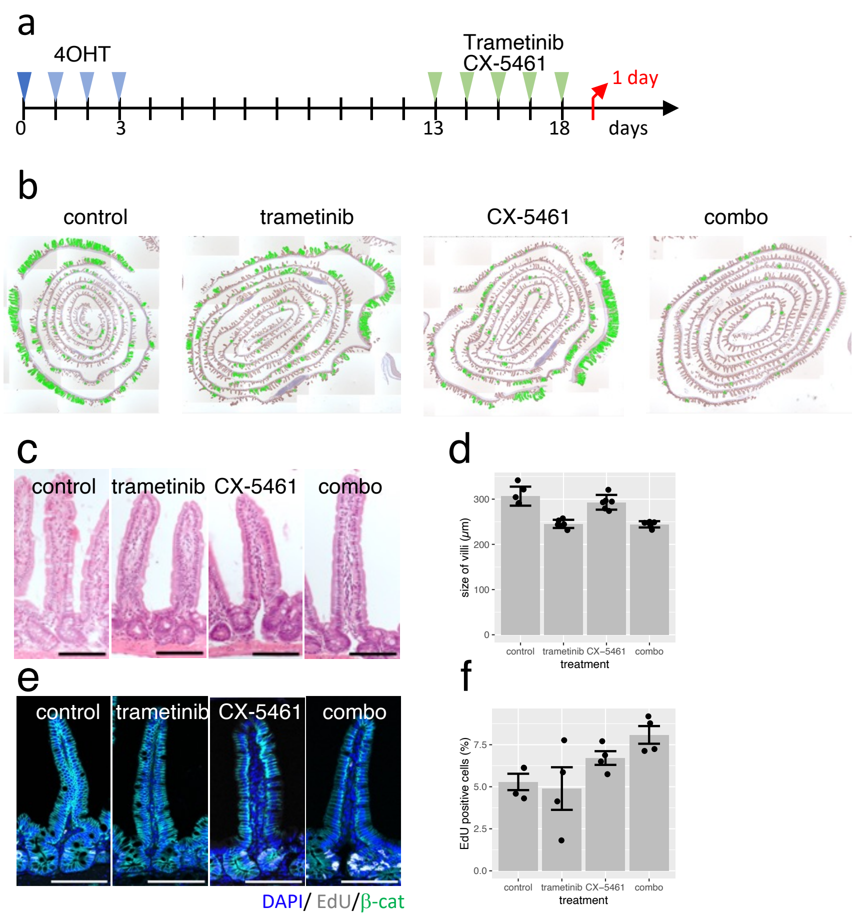

Supplementary Figure 4. Evaluation of the effects of trametinib and CX-5461 in a mouse model of intestinal tumor. a. Experimental design. Recombination by Lgr5-CreERT2 was induced via the subcutaneous injection of 4OHT at 40 mg/kg on day 0 (dark blue arrow) and 20 mg/kg on days 1–3 (light blue arrows). Dimethyl sulfoxide (DMSO), trametinib (1 mg/kg), and CX-5461 (30 mg/kg) were administered daily from days 13 to 18 (green arrows). Mice were sacrificed, and intestinal tumors were analyzed on day 19 (red bar). **b.** Evaluation of tumor burden. Formalin-fixed intestinal Swiss roles were stained with the anti β-catenin antibody, and tiling images were scored using automated measurement modules. Tumor regions are shown in green. c,d. Evaluation of normal intestine in trametinib- and CX-5461-treated *Apc*$^{s/s}$, *Kras*$^{LSL\text{-}G12D+/-}$*,Lgr5-CreERT2* mice. Representative HE images of mouse intestine treated with the indicated drugs are shown (c). Bar =100 μm. Villi size is shown in the barplot (mean ± SD, N=4) (d). Statistical significance was assessed using a two-sided Welch's t-test. **e,f.** EdU incorporation in trametinib-treated mouse intestine.

Representative images of incorporated EdU (gray), β-catenin (green), and nuclei (blue) in normal intestine from control and trametinib-treated mice are shown. (e) Bar =100 μm. The percentage of EdU-positive nuclei per villus is shown in the barplot (mean ± SD, N=4) (f). Statistical significance was assessed using a two-sided Welch’s t-test.

a

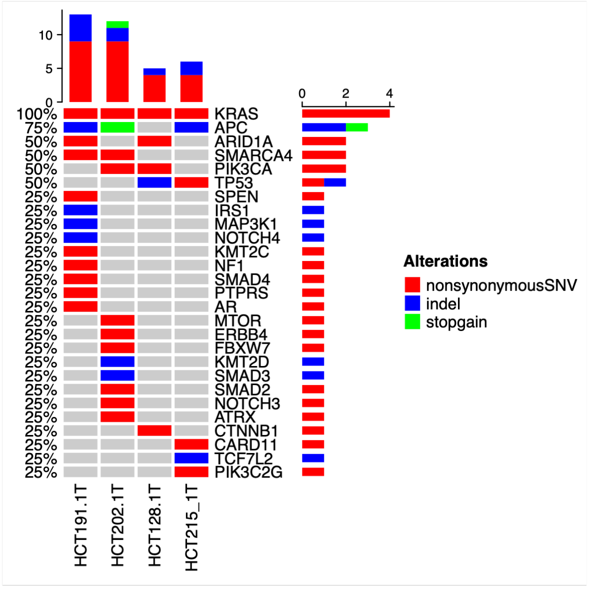

b

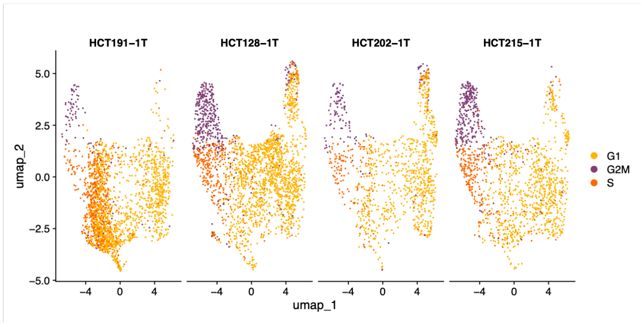

Supplementary Figure 5. Characterization of CRC PDOs with KRAS G12C mutation. a. Genomic profiling of PDOs. Overview of PDOs established from patients with CRC harboring the KRAS G12C mutation. Top 100 most frequently mutated genes in CRC [1] identified in the organoid sets are listed and their mutation frequencies are shown in the left row. Top and right panels show the numbers of mutated PDOs and genes, respectively. Mutation types are indicated in the right panel. **b.** Cell cycle scoring of PDOs. UMAP of PDOs colored according to the cell cycle score is shown.

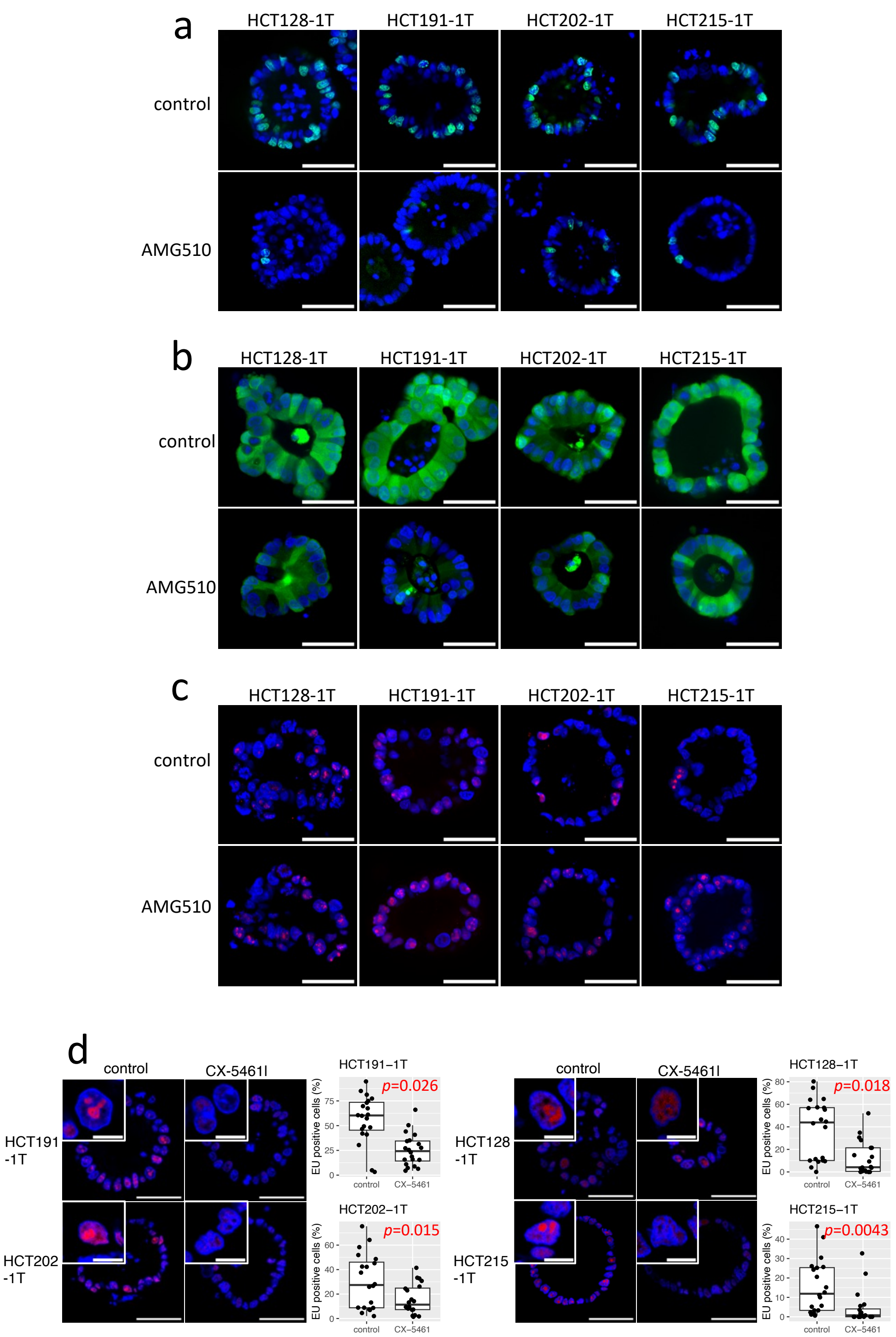

Supplementary Figure 6. Images of AMG510-treated KRAS G12C-mutated CRC organoids. a-c. Organoids were treated with 10 nM AMG510 for 24 h, and the incorporation of 5-ethynyl-2'-deoxyuridine (EdU) (a) and O-propargyl-puromycin (OPP) (b) were visualized using the Click reaction. Fibrillarin was

visualized by immunofluorescent staining (c). Representative images quantifying the percentage of positive cells are shown in Fig. 6a to c. Bar = 50 ed EU incorporation in CX-5461-treated CRC organoids. Representative images of EU (red) and nuclei (blue) in control and CX-5461-treated organoids are shown. High magnification views of the white boxed regions are shown in the insets. Bar = 50µm, Bar in inset = 10µm. The percentage of EU-positive nuclei per organoid is shown in the boxplot. Data of 20 PDOs obtained from four independent experiments are shown. *p*-values were determined by two-sided Welch's *t*-test.

Supplementary Table 1. Differential expression of ground state markers in the C1 cluster.

	p_val	avg_log2FC	pct.1	pct.2	p_val_adj
LEFTY1	1.89E-202	3.10384337	0.587	0.095	2.91E-198
RPL34	1.90E-118	0.54256129	1	1	2.92E-114
RPLP1	1.07E-107	0.39458253	1	1	1.65E-103
RPL10	1.93E-83	0.45136182	1	0.999	2.97E-79
PABPC1	2.23E-80	0.73773889	0.963	0.914	3.42E-76
SLC12A2	2.05E-24	0.37790496	0.85	0.751	3.15E-20
VMP1	2.15E-20	0.51202582	0.594	0.42	3.30E-16
TXNIP	2.57E-10	0.3960925	0.72	0.697	3.95E-06
PDGFA	7.88E-10	1.0994807	0.11	0.052	1.21E-05
ARID5B	2.08E-08	0.44314445	0.346	0.251	0.00031949

Differential expression analysis was performed using the Wilcoxon rank-sum test implemented in Seurat, with p-values adjusted using the Benjamini–Hochberg method.

Supplementary Table 2. Differential expression of ribosomal genes in the C1 cluster.

	p_val	avg_log2FC	pct.1	pct.2	p_val_adj
RPL13A	1.47E-138	0.50086863	1	1	2.26E-134
RPS18	5.01E-97	0.34636505	1	1	7.70E-93
RPL7A	2.65E-72	0.38978246	1	1	4.07E-68
RPL36A	1.01E-66	0.42787462	1	1	1.55E-62

Differential expression analysis was performed using the Wilcoxon rank-sum test implemented in Seurat, with p-values adjusted using the Benjamini–Hochberg method.

Supplementary Table 3. Clinical information of PDOs derived from stage IV CRC.

Case No	organoid ID	gender	age range	location*	primary/metastasis/recurrent	pre-operative chemotherapy
HCT191	HCT191-1T	female	60-65	C	primary	mFOLFOX6 + BV x22
HCT128	HCT128-1T	male	70-75	A	primary	none
HCT202	HCT202-1T	male	55-60	A	primary	none
HCT215	HCT215-1T	male	60-65	Ra	primary	none

C-cecum; A-ascending colon; Ra-rectum
mFOLFOX6-fluorouracil (5-FU) and leucovorin with oxaliplatin; BV-bevacizumab

Supplementary Table 4. primers used for mouse genotyping.

allele	primer ID	primer sequence
APC580S	APC43	5'-GTTCTGTATCATGGAAAGATAGGTGTC-3'
	APC45	5'-CACTCAAAACGCTTTTGAGGGTTGATTC-3'
KrasG12D	Kras_1lox_2	5'-CTCTTGCCTACGCCACCAGCTC-3'
	Kras_1lox_3	5'-AGCTAGCCACCATGGCTTGAGTAAGTCTGCA-3'
Lgr5-CreERT	Lgr5-GT1	5'-TCTACAGGCTCCCTGCTCTCTGCTC-3'
	EGFP-1	5'-GTGCTGCTTCATGTGGTCGGGGTAG-3'

1 Yaeger, R. *et al.* Clinical Sequencing Defines the Genomic Landscape of Metastatic Colorectal Cancer. *Cancer Cell* **33**, 125-136 e123 (2018). https://doi.org/10.1016/j.ccell.2017.12.004
