# Supplementary material for: Ribosome biogenesis as a potential therapeutic target in KRAS mutant colorectal cancer
Source: Nat Commun. 2025 Dec 27;17:1224. doi: 10.1038/s41467-025-67979-9 (PMC12864942; doi:10.1038/s41467-025-67979-9)
Supplement: Supplementary file 2 — Reporting Summary [file 41467_2025_67979_MOESM2_ESM.pdf]

Corresponding author(s): Ryoji Yao
Last updated by author(s): Aug 9, 2025

Reporting Summary

Nature Portfolio wishes to improve the reproducibility of the work that we publish. This form provides structure for consistency and transparency in reporting. For further information on Nature Portfolio policies, see our Editorial Policies and the Editorial Policy Checklist.

Statistics

For all statistical analyses, confirm that the following items are present in the figure legend, table legend, main text, or Methods section.

n/a	Confirmed	
☐	☒	The exact sample size (*n*) for each experimental group/condition, given as a discrete number and unit of measurement
☐	☒	A statement on whether measurements were taken from distinct samples or whether the same sample was measured repeatedly
☐	☒	The statistical test(s) used AND whether they are one- or two-sided<br>*Only common tests should be described solely by name; describe more complex techniques in the Methods section.*
☒	☐	A description of all covariates tested
☒	☐	A description of any assumptions or corrections, such as tests of normality and adjustment for multiple comparisons
☐	☒	A full description of the statistical parameters including central tendency (e.g. means) or other basic estimates (e.g. regression coefficient) AND variation (e.g. standard deviation) or associated estimates of uncertainty (e.g. confidence intervals)
☐	☒	For null hypothesis testing, the test statistic (e.g. *F*, *t*, *r*) with confidence intervals, effect sizes, degrees of freedom and *P* value noted<br>*Give P values as exact values whenever suitable.*
☒	☐	For Bayesian analysis, information on the choice of priors and Markov chain Monte Carlo settings
☒	☐	For hierarchical and complex designs, identification of the appropriate level for tests and full reporting of outcomes
☒	☐	Estimates of effect sizes (e.g. Cohen's *d*, Pearson's *r*), indicating how they were calculated

Our web collection on statistics for biologists contains articles on many of the points above.

Software and code

Policy information about availability of computer code

Data collection	Organoids and cell images were collected by CV8000 Imaging System (Yokogawa) and LSM880 Confocal Microscope (Zeiss). Histological and immunostaining images were collected by LSM880 Confocal Microscope (Zeiss) and Axio Imager2 (Zeiss). Immunoblotting images were collected by ChemiDoc XRS+ system (Bio Rad). qRT-PCR data were collected from LightCycler System (Roche). scRNA-seq data generated in this study was collected by Illumina NextSeq 500 platform(Illumina).
Data analysis	n/aFor scRNA-seq analysis, Cell Ranger (v.6.1.1) and Seurat was used to collapse gene matrix files and to assess the statistical significance of differentially expressed genes. Gene Set Enrichment Analysis was performed using the fgsea package with nperm=1000 (https://bioconductor.org/packages/release/bioc/html/fgsea.html). Tidy format gene sets were retrieved using msigdbr package (https://cran.r-project.org/web/packages/msigdbr/vignettes/msigdbr-intro.html), and the rnk file was generated using wilcoxauc function in presto package (https://rdrr.io/github/immunogenomics/presto/man/wilcoxauc.html). Mass spectrometry data were processed using MaxQuant software (version 1.5.1.2 or 1.6.3.30). Additional software used: Microsoft Excel (version:16.99.2), Graphpad Prism 9 (Graphpad), QuPath v.0.1.3 (Queen's University, Belfast, Northern Ireland), Fiji (NIH), Zen blue (Zeiss), RStudio (4.4.1).

For manuscripts utilizing custom algorithms or software that are central to the research but not yet described in published literature, software must be made available to editors and reviewers. We strongly encourage code deposition in a community repository (e.g. GitHub). See the Nature Portfolio guidelines for submitting code & software for further information.

Data

Policy information about availability of data

All manuscripts must include a data availability statement. This statement should provide the following information, where applicable:

- Accession codes, unique identifiers, or web links for publicly available datasets
- A description of any restrictions on data availability
- For clinical datasets or third party data, please ensure that the statement adheres to our policy

The RNA-seq data generated in this study have been deposited in the Gene Expression Omnibus (GEO) database under accession code GSE264485 [https://www.ncbi.nlm.nih.gov/geo/query/acc.cgi?acc=GSE264485]. The raw mass spectrometry (MS) data generated in this study have been deposited in the ProteomeXchange Consortium (https://www.proteomexchange.org/) via the Jpost partner repository under accession ID PXD052914/JPST003164 [https://repository.jpostdb.org/entry/JPST003164.0].

Research involving human participants, their data, or biological material

Policy information about studies with human participants or human data. See also policy information about sex, gender (identity/presentation), and sexual orientation and race, ethnicity and racism.

Reporting on sex and gender	Patient derived organoids were established from three males and one female. No sex related analysis was performed due to the low number of patients.
Reporting on race, ethnicity, or other socially relevant groupings	This study do not involve any race, ethnicity, or other socially relevant groupings considered for the analysis.
Population characteristics	The population consists of colorectal cancer patients aged between 55 and 75 years with a KRAS G12C mutation. Among them, one patient received chemotherapy, while the others did not.
Recruitment	Participants were colorectal cancer patients who underwent surgery at the Cancer Institute Hospital of the Japanese Foundation for Cancer Research (JFCR). Written informed consent was obtained from all individuals prior to inclusion in the study.
Ethics oversight	This study was approved by the institutional review board of the Cancer Institute Hospital of the Japanese Foundation for Cancer Research (approval number: 2013-1105) and was conducted in accordance with the Declaration of Helsinki.

Note that full information on the approval of the study protocol must also be provided in the manuscript.

Field-specific reporting

Please select the one below that is the best fit for your research. If you are not sure, read the appropriate sections before making your selection.

☒ Life sciences ☐ Behavioural & social sciences ☐ Ecological, evolutionary & environmental sciences

For a reference copy of the document with all sections, see nature.com/documents/nr-reporting-summary-flat.pdf

Life sciences study design

All studies must disclose on these points even when the disclosure is negative.

Sample size	The sample size was determined based on previous studies and statistical power analysis to ensure adequate power to detect a significant effect
Data exclusions	Low quality cells were excluded in scRNA-seq as described in the Methods.
Replication	In the patient derived organoid experiments, each measurement was performed at least three times. For mouse experiments, a minimum of three animals were included. The number of replicates for each experiment is described in the figure legends.
Randomization	Mice were randomly assigned to treatment and control groups.
Blinding	Blinding was not necessary for this study due to the objective nature of the outcome measurements.

Reporting for specific materials, systems and methods

We require information from authors about some types of materials, experimental systems and methods used in many studies. Here, indicate whether each material, system or method listed is relevant to your study. If you are not sure if a list item applies to your research, read the appropriate section before selecting a response.

Materials & experimental systems

n/a	Involved in the study
☐	☒ Antibodies
☐	☒ Eukaryotic cell lines
☒	☐ Palaeontology and archaeology
☐	☒ Animals and other organisms
☐	☒ Clinical data
☒	☐ Dual use research of concern
☒	☐ Plants

Methods

n/a	Involved in the study
☒	☐ ChIP-seq
☒	☐ Flow cytometry
☒	☐ MRI-based neuroimaging

Antibodies

Antibodies used	anti-fibrillarin (ab5821; Abcam)1:500<br>anti-CD44v6 (MCA1967; BioRad) 1:400<br>anti-b-catenin (610154, BD Biosciences) 1:1000<br>anti-PABP1 (10970-1, Proteintech) 1:1000<br>anti-RPL13A (14633-1-AP, Proteintech) 1:1000<br>anti-RPL23 (16086-1-AP, Proteintech) 1:1000<br>anti-b-actin (A1978, Sigma) 1:1000
Validation	Antibodies used in this study were validated by the manufacturer as stated on the manufacturers website. Manufacturer7s validation statements are described on the following websites:<br>anti-fibrillarin (ab5821; Abcam) https://www.abcam.co.jp/products/primary-antibodies/fibrillarin-antibody-nucleolar-marker-ab5821.html<br>anti-CD44v6(MCA1967;BioRad)https://www.bio-rad-antibodies.com/monoclonal/mouse-cd44v6-antibody-9a4-mca1967.html?f=purified&evCntryLang=JP-JA&ROUTEID=.appserver2&JSESSIONID_STERLING=adummyvalue<br>anti-b-catenin (610154, BD Biosciences) https://www.bdbiosciences.com/ja-jp/products/reagents/microscopy-imaging-reagents/immunofluorescence-reagents/purified-mouse-anti-catenin.610154?tab=product_details<br>anti-PABP1 (10970-1, Proteintech) https://www.ptglab.co.jp/products/PABPC1,PABP-Antibody-10970-1-AP.htm<br>anti-RPL13A (14633-1-AP, Proteintech) https://www.ptglab.co.jp/products/RPL13A-Antibody-14633-1-AP.htm<br>anti-RPL23 (16086-1-AP, Proteintech) https://www.ptglab.co.jp/products/RPL23-Antibody-16086-1-AP.htm<br>anti-b-actin (A1978, Sigma) https://www.sigmaaldrich.com/JP/ja/product/sigma/a1978?srsltid=AfmBOoomIVhs_NZDuqIWvrE5o6Mjk-w9S4Y2gBJYEjNAqyQ3wRWBLGBP

Eukaryotic cell lines

Policy information about cell lines and Sex and Gender in Research

Cell line source(s)	Human patient derived organoids were established in this study.<br>Noggin expressing L1 cell was established in our laboratory.
Authentication	Authentication of the cell lines was performed based on genetic mutation analysis, including the identification of KRAS mutations.
Mycoplasma contamination	Mycoplasma contamination was routinely tested and confirmed to be negative.
Commonly misidentified lines<br>(See ICLAC register)	None of the misidentified lines were used in this study.

Animals and other research organisms

Policy information about studies involving animals; ARRIVE guidelines recommended for reporting animal research, and Sex and Gender in Research

Laboratory animals	ApcS/S(Shibata et al. Science 1997), KrasLSL-G12D/+(Johnson et al. Nature 2001), Lgr5-CreERT2(Huch et al., Naure 2013) mice were previously described. All mouse strains were generated in a C57BL/6 background. 10week-old mice were used in all experiments. The mice were maintained in 12h light/12h dark cycle. The housing temperature and humidity were 24 C and 50%, respectively.
Wild animals	This study does not involve wild animals.
Reporting on sex	Sex-based analysis was not performed in this study.
Field-collected samples	This study did not involve sample collected in the field.
Ethics oversight	All animal experiments were conducted in accordance with institutional guidelines and approved by the Animal Care and Use Committee of Japanese Foundation of Cancer Institute, Japan.

Note that full information on the approval of the study protocol must also be provided in the manuscript.

Clinical data

Policy information about clinical studies

All manuscripts should comply with the ICMJEguidelines for publication of clinical research and a completedCONSORT checklist must be included with all submissions.

Clinical trial registration	*Provide the trial registration number from ClinicalTrials.gov or an equivalent agency.*
Study protocol	*Note where the full trial protocol can be accessed OR if not available, explain why.*
Data collection	*Describe the settings and locales of data collection, noting the time periods of recruitment and data collection.*
Outcomes	*Describe how you pre-defined primary and secondary outcome measures and how you assessed these measures.*

Plants

Seed stocks	n/a
Novel plant genotypes	n/a
Authentication	n/a
